# Early palliative care versus standard care in haematologic cancer patients at their last active treatment: study protocol of a feasibility trial

**DOI:** 10.1186/s12904-020-00561-w

**Published:** 2020-04-22

**Authors:** Silvia Tanzi, Stefano Luminari, Silvio Cavuto, Elena Turola, Luca Ghirotto, Massimo Costantini

**Affiliations:** 1 Palliative Care Unit, Azienda USL-IRCCS di Reggio Emilia, Viale Risorgimento 80, 42123 Reggio Emilia, Italy; 2 Clinical and Experimental Medicine PhD program, University of Modena and Reggio Emilia, Modena, Italy; 3 Haematology Department, Azienda USL-IRCCS di Reggio Emilia, Reggio Emilia, Italy; 4 Clinical Trials an Statistics Unit, Infrastructure Research and Statistic, Azienda USL-IRCCS di Reggio Emilia, Reggio Emilia, Italy; 5 Qualitative Research Unit, Azienda USL-IRCCS di Reggio Emilia, Reggio Emilia, Italy; 6 Scientific Directorate, Azienda USL-IRCCS di Reggio Emilia, Reggio Emilia, Italy

## Abstract

**Background:**

Patients with advanced haematological malignancies suffer from a very high symptom burden and psychological, spiritual, social and physical symptoms comparable with patients with metastatic non-haematological malignancy. Referral to palliative care services for these patients remains limited or often confined to the last days of life. We developed a palliative care intervention (PCI) integrated with standard haematological care. The aim of the study was focussed on exploring the feasibility of the intervention by patients, professionals and caregivers and on assessing its preliminary efficacy.

**Methods/design.**

This is a mixed-methods phase 2 trial.

The Specialist Palliative Care Team (SPCT) will follow each patient on a monthly basis in the outpatient clinic or will provide consultations during any hospital admission. SPCT and haematologists will discuss active patient issues to assure a team approach to the patient’s care.

This quantitative study is a monocentric parallel-group superiority trial with balanced randomisation comparing the experimental PCI plus haematological standard care versus haematological standard care alone.

The primary endpoint will calculate *on* adherence to the planned PCI, measured as the percentage of patients randomised to the experimental arm who attend all the planned palliative care visits in the 24 weeks after randomisation.

The qualitative study follows the methodological indications of concurrent nested design and was aimed at exploring the acceptability of the PCI from the point of view of patients, caregivers and physicians.

**Discussion:**

In this trial, we will test the feasibility of an integrated palliative care approach starting when the haematologist decides to propose the last active treatment to the patient, according to his/her clinical judgement. We decided to test this criterion because it is able to intercept a wide range of patients’needs. The feasibility of this approach requires that we enrol at least 60 patients and that more than 50% of them be followed by the palliative care team for at least 24 weeks.

The trial will include integrated qualitative data analysis; to give essential information on feasibility and acceptability.

**Trial registration:**

ClinicalTrials.gov: NCT03743480 (November 16, 2018).

## Background

The most recent World Health Organization (WHO) definition of palliative care advocates that palliative care principles “…should be applied as early as possible in the course of any chronic, ultimately fatal illness” [1]. The difference to the previous WHO vision is substantial [2], as the earlier definition recommended palliative care to patients not responsive to curative therapy, limiting its role to the last period of life.

Evidence about the effectiveness of an early integration of palliative care has emerged in recent years for patients with solid tumours. A recent Cochrane systematic review identified seven eligible randomised clinical trials (RCTs) comparing the effects of early palliative care interventions versus standard cancer care on quality of life, depression, symptom intensity, and survival among advancer cancer patients [3]. The results of this review suggested that early palliative care had significantly beneficial effects on quality of life, with a standardised mean difference (SMD) of 0.27 (95% confidence interval (CI) 0.15 to 0.38), and on symptom intensity, with a SMD of − 0.23 (95% CI − 0.35 to − 0.10), among patients with advanced cancer. Effects on mortality and depression remained uncertain [4]. The results of qualitative studies performed in different countries suggested that the early integration of specialised palliative care is well accepted by patients, relatives and, to a lesser extent, oncologists [5–8].

Patients with advanced haematological malignancies suffer from a very high symptom burden and psychological, spiritual, social and physical symptoms comparable with patients with metastatic non-haematological malignancy [9–11]. During the last 30 days of life, haematological patients are more frequently admitted to hospital settings, emergency departments and high-care wards and receive more aggressive treatments and more chemotherapy or biologically active treatments than patients with advanced solid tumours [12].

Conversely, referral to palliative care services for haematologic patients remains limited or often confined to the last days of life [13, 14]. Haematologic specialists show resistance to involving a palliative care service because of the perceived contrast between ongoing active treatments and palliative care, the latter being identified as terminal care [15].

In agreement with the new WHO vision [1], the evidence from studies performed in patients with solid tumours and haematologic patients’ symptom burden suggests that an earlier and integrated provision of specialised palliative care has the potential to improve their quality of life and reduce resource consumption through effective management of psychological and physical symptoms, appropriate relationships, effective communication and support in decision-making.

The call for an effective model of assistance integrating specialised palliative care and haematologic services is strong [16], although there is no agreement about what the best approach is. To some authors, palliative care should be integrated earlier in the trajectory of the advanced disease [17, 18], while others suggest an early integration according to patients’ needs [19, 20] or when requested by the haematologist [21]. In some centres, integration of palliative care is assessed for patients undergoing stem cell transplantation [22].

A major challenge in designing a palliative care intervention that is early integrated with onco-haematological care is identifying the best timing for starting an integrated intervention.

The evidence of the effectiveness of an early integration of palliative care with onco-haematological care is poor and is based on few observational studies [10, 11, 23–26]. Only one randomised clinical trial assessed the effectiveness of a palliative care intervention (PCI) in patients hospitalised for haematopoietic stem cell transplantation [27]. The results showed a smaller decrease in quality of life for patients randomised to receive PCI 2 weeks after transplantation.

Following the WHO vision, we developed a PCI integrated with standard haematological care. This pilot study was primarily focused on assessing the feasibility of the PC intervention. Secondary aims include exploring its acceptability by patients, professionals and caregivers and collecting preliminary information on its effectiveness, which will be potentially useful for designing a randomised phase 3 trial. In this article, we describe the protocol of the study.

## Methods

### Trial design

According to the Medical Research Council framework for complex interventions [28, 29], this is a mixed-methods phase 2 trial on early integration of palliative care in patients with advanced haematological malignancies.

This quantitative study is a monocentric parallel-group superiority trial with balanced randomisation (1:1) comparing the experimental PCI plus haematological standard care versus haematological standard care alone (control arm).

The qualitative study follows the methodological indications of concurrent nested design [30], as both qualitative and quantitative data are collected during the same stage. The qualitative study will deepen the perceptions of patients, family members and physicians involved in the early integrated PCI.

### Aims

#### Primary aim

The primary aim is to determine whether a specialised PCI integrated with standard haematological care is feasible in terms of adherence to the planned PCI.

#### Secondary aims

Quantitative secondary aims are to obtain Patient Reported Outcomes Measures (PROMS) estimates to assess the potential effectiveness of the intervention and to inform the sample size calculations for a future large-scale trial. More specifically, secondary aims include the following assessments in the 24 weeks after enrolment:

• compliance with the assessment of quality of life;

• the main dimensions of quality of life measured with the Palliative Care Outcome Scale questionnaire (POS);

• the severity of physical and psychological symptoms measured with the Edmonton Symptom Assessment Scale questionnaire (ESAS);

• The severity of symptoms of anxiety and depression measured with the Hospital Anxiety and Depression Scale questionnaire (HADS);

• the functional status of the patient measured with the Eastern Cooperative Oncology Group scale (ECOG Performance Status).

Qualitative secondary aims are to explore the acceptability, perceived benefits and concerns, and strengths and weaknesses of the PCI from the point of view of interviewed patients, caregivers and physicians.

### Eligibility criteria

#### Inclusion criteria

- histologically or cytologically confirmed incurable haematological malignancy;
- age 18 years or more.
- life expectancy more than 1 month, according the clinical judgement of both the haematologist and the palliative care physician;
- The patient will start the last potentially active treatment (chemotherapy or immunotherapy) as established by the haematological team. Another active treatment could follow this treatment according to evidenced best practice and/or the development of novel haematologic therapy. Agreement on the new treatment between referenced physicians is guaranteed.
- performance status of 0–3, according to the Eastern Cooperative Oncology Group (ECOG);
- ability to understand, read and fill in questionnaires in Italian;
- signed the written informed consent form.

#### Exclusion criteria

- patient without a caregiver;
- any physical, psychological or psychiatric condition that, in the opinion of the clinical team, makes participation to the study not appropriate.

### Setting

Participants will be recruited from the Haematology Department of the Arcispedale Santa Maria Nuova of Reggio Emilia, Italy.

The Haematology division is located in the Santa Maria Nuova hospital, Centro Onco Ematologico of Reggio Emilia. It includes inpatient and outpatient services. The inpatient service consists of 16 beds, 6 of which are used for bone marrow transplantation (low microbial charge). The outpatient service consists of 4 rooms with a total of 6 beds and 3 armchairs and 10 rooms for medical examinations. Medical staff is made up of 14 haematologists organised by main area of haematology: inpatient and transplant, lymphomas, myeloproliferative disorders, and acute leukaemia. During 2018, there were 272 hospital admissions and 39,479 ambulatory visits. The unit is certified by Jaice for bone marrow transplantation (autologous and allogenic) and is also certified for phase I trials.

The PCI is provided by the hospital Palliative Care Unit (PCU), integrated for the care of patients and relatives with severe psychological suffering with the hospital Psycho-Oncology Unit. The PCU can be classified as a specialised hospital-based unit with no beds [31]. At present, it includes three senior physicians (one in advanced palliative care training) and two advance practice nurses, with a remit of specialist consultations in wards and in a clinic for advanced outpatients and their relatives.

### Screening and informed consent

**Fig. 1:**
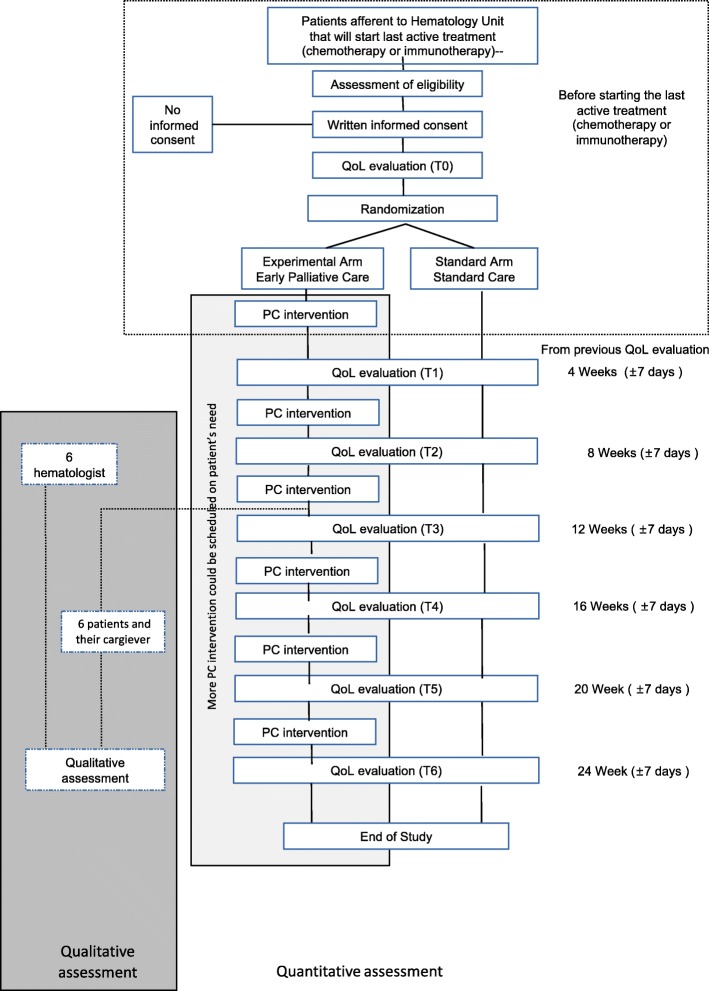
Flow chart EPC-EMA 1

All study procedures are described in Fig. 1.

Haematologists and palliative care physicians identify potentially eligible patients during weekly case discussions in the Haematology Department.

We will register all potentially eligible patients and reasons for no accruement, patients who were asked to participate in the study, and patients who did not agree to participate.

A haematologist will give to each eligible patient a description of the study and will ask them to participate in the study.

Patients who agree to participate in the trial will be asked to sign the written informed consent.

To achieve adequate patient enrolment and reach the target sample size, the enrolment rate will be constantly monitored. To this aim, contacts with physicians will take place on a weekly basis.

### Randomisation

All patients who agree to participate in the study will be randomly assigned to either the experimental or the control group (1:1 ratio) according to a computer-generated randomisation list created using permuted blocks of random (undisclosed) sizes and stratified by site (if other centres will join patient recruitment).

The allocation sequence will be generated by the Infrastruttura Ricerca e Statistica (I-RS) of the Azienda USL-IRCCS. Investigators involved in patient recruitment will call by telephone the central randomisation centre (I-RS), which will assign the patient a unique code and communicate the arm of allocation. The randomisation code will not be released before patient enrolment to ensure allocation concealment.

### Endpoints

#### Primary endpoint

• Adherence to the planned PCI, measured as the percentage of patients randomised to the experimental arm who attend all the planned palliative care visits in the 24 weeks after randomisation.

#### Secondary endpoints

- Compliance with the assessment of quality of life for each questionnaire (POS, ESAS and HADS) at the six planned times;
- Assessment of quality of life measured with the Palliative Care Outcome Scale questionnaire (POS);
- The severity of physical and psychological symptoms measured with the Edmonton Symptom Assessment Scale questionnaire (ESAS);
- The severity of symptoms of anxiety and depression measured with the Hospital Anxiety and Depression Scale questionnaire (HADS);
- The functional status of the patient measured with the Eastern Cooperative Oncology Group scale (ECOG Performance Status).

All questionnaires will be proposed to patients (control/experimental arm) for self-assessment by two trained researchers at the following time points: immediately after giving consent (t0), 4 weeks (± 7 days) after t0 (t1), 8 weeks (± 7 days) after t0 (t2), 12 weeks (± 7 days) after t0 (t3), 16 weeks (± 7 days) after t0 (t4), 20 weeks (± 7 days) after t0 (t5) and 24 weeks (± 7 days) after t0 (t6).

Self-assessment will be the standard procedure. If this is not possible, patients will be assisted by a research physician. Assessment by other professionals or caregivers is not allowed.

Baseline evaluation (t0) will be carried out in hospital. To reduce deviation from the protocol and the dropout rate, evaluations t1-t6 could be performed in hospital or at the patient’s home, depending on the patient’s preferences.

Four questionnaires will be used in this study:

- Palliative Care Outcome Scale (POS – version 2) in its Italian-validated version [32]. POS is a widely used questionnaire with a brief outcome measure. It is commonly used in patients with advanced illnesses in different settings: inpatient, outpatient, and community. POS comprises 10 items, including physical and psychological symptoms, spiritual and emotional dimensions, communication with patients and families, and practical concerns related to disease stage. Each of the 10 items is scored with a Likert scale ranging from 0 to 4. POS also includes an open optional question to list the patient’s main concerns.
- The Edmonton Symptom Assessment Scale (ESAS) in its Italian-validated version [33]. ESAS asks respondents to rate the severity of 10 common symptoms (pain, fatigue, nausea, depression, anxiety, drowsiness, shortness of breath, appetite, sleep, and feeling of well-being) during the previous 24 h. It has been found to be valid and reliable in cancer populations.
- The Hospital Anxiety and Depression Scale (HADS) in its Italian-validated version [34]. HADS is a self-assessed 14-item questionnaire that has been tested in cancer patients. It has two 7-item subscales assessing depression and anxiety in the preceding week. The scale is considered appropriate for cancer patients because of the lack of items regarding somatic symptoms, which can confound the identification of psychiatric issues. The format consists of four responses (range 0 to 3) that quantify the degree to which an emotion is experienced by the patient. The score on each subscale ranges from 0 to 21, and a score greater than 11 is consistent with definitive depression or anxiety. A score of less than 7 is normal, and a score of 8–10 is considered borderline for depression and anxiety.
- The Eastern Cooperative Oncology Group (ECOG) Performance Status [35], a widely used scale to evaluate and measure the functional status of a patient.

### Data collection and management

We will collect Information on the use of health care services including referral to hospice, hospital admissions,emergengy department visits and the date and place of death. In particular we will collect frequency and date of palliative care visits in each arm, type, setting and timing of all chemotherapy, number of hospitalizations, emergency visits and use of hospice services.

Data will be collected on an electronic Case Report File (eCRF) management system (Smarty Ver.3.85– Sinfo S.R.L), validated and handled in accordance to local and European regulatory requirements.

### The qualitative assessment

A consecutive series of six patients enrolled in the experimental arm who attend at least three visits in the PC outpatient clinic and their family members will be asked to participate in the qualitative assessment. This assessment will also be proposed to a sample of six physicians involved in the care of recruited patients, selected among those who follow the highest numbers of patients. Patients, family members, and physicians who agree to participate in the qualitative study will be asked to sign the specific written informed consent for the interview and to give explicit permission to be audio-recorded.

Information will be gathered through individual semi-structured interviews [36]. The prompts are defined in advance and listed in three topic guides [37] whose responsibility is up to a team member, expert in qualitative evaluation in palliative care (Table 1). Researchers will explore the acceptability of the intervention giving the opportunity to the receivers (patients and family members) and the haematologists, involved in early integrated palliative care, to tell their experience. Given the intervention’s characteristics discussed below, while the semi-structured interviews administered to patients and family members will focus on perceived benefits and concerns of the early PC intervention, the physicians’ interviews will address the strengths and weaknesses of the intervention with reference to the respondents’ views on patient and family caregiver experience.

**Table 1:** Semi-structure interview topic guides with prompts

Topic	Patients	Family members	Physicians
Early integration of PC	Could you please tell me what you thought when the haematologist proposed you this intervention? Did you talk with anyone about it? How did you experience it?	Could you please tell me what you thought when the haematologist proposed to your loved-one this intervention?	Could you please tell me what you thought about this intervention when you heard about it?
Relationship	Could you please tell me how is the relationship with the hematologist? And what about the palliativist?	Could you please tell me what is your relationship with the hematologist? And what about the palliativist?	How is your relationship as a physician with the patients in the study?
Perceived benefits/strengths	Regarding your participation in this study, could you please tell me what was good for you? What can be the positive aspects of this?	Regarding the participation in this study, could you please tell me what was good for you as caregiver? What can be the positive aspects of this?	Regarding this study, could you please tell me what the strengths of this intervention are, according to your opinion? If it impacted on your usual job, how did it do?
Concerns/weaknesses	Could you please tell me if you had concerns about the intervention? What can be the negative aspects of this?	Could you please tell me if you had concerns about the intervention? What can be the negative aspects of this?	Regarding this study, could you please tell me what the weaknesses of this intervention are?
Feelings	In general, could you please tell me how you felt during this study? Is there any example you would like to share? What about the support you received?	In general, could you please tell me how you felt during this study? Is there any example you would like to share? What about the support you received? And what about the support your loved-one received?	As professional caregiver, could you please tell me how you felt? How did you experience the relationship with the palliativist? And with the patients and their family members?
Decision-making and advance care planning	Could you please tell me how you made the decisions this intervention required? What did you think when you have been involved in the advance care planning?	Could you please tell me how the decision-making process went? What did you think when you have been involved in the advance care planning of your loved-one?	Could you please tell me your opinion about the decision-making process with patients and caregivers during the intervention? How did it go? What are you opinions/prerogatives about/in advance care planning?

Anonymity and non-traceability criteria will be duly presented to all interviewees. The interviewers will be two researchers with expertise in palliative care and basic knowledge of the intervention but who will not be involved in its implementation.

Training of all researchers involved in the study will be carried out before the enrolment of any patient to guarantee the study integrity.

### The intervention offer

Haematologists will propose that a patient participate in a study that will include early PC intervention integrated with standard haematological care. To standardise the proposal, a wording will be suggested to all the haematologists as requested in a previous focus group between haematologists and palliative care professionals in which haematologists addressed the difficulty of using words such as *palliative care* or *palliative care intervention* because perceived by patients as synonymous with *terminal care*. The standardised passage will be,

“I propose you participate in a study in which you will also be assisted by the staff of the palliative care unit. Palliative care addresses all quality-of-life domains, and we have been collaborating with this unit for several years. However, there are no studies anywhere in the world addressing the efficacy of this integrating model of assistance. We would highly appreciate your participation in this study. Even if you decide not to participate in the study, we will continue to assist you according to the best standard of care.”

### The PCI in the experimental arm

The initial assessment of patients enrolled in the experimental arm will occur in the outpatient clinic or in the ward as a consultation before the beginning of the last active treatment. The Specialist Palliative Care Team (SPCT) will follow each patient on a monthly basis in the outpatient clinic or will provide consultations during any hospital admission. SPCT and haematologists will discuss active patient issues to assure a team approach to the patient’s care. We will also consider family meetings to improve communication among the patient, family members and health professionals in which we will share the patient’s status, goals of care and current plans. Patients will be followed until death, referral to PC community teams, refusal, or other reasons.

More specifically, the goals of integrating palliative care earlier during the disease include the following:

- Specific attention to individual preferences for information, including patient understanding/awareness of the prognosis;
- improved physical and psychological symptom detection and management;
- a continuous explanation of treatment goals and support for patient decision-making;
- elements of advance care planning, progressively introduced, according to the patients’ wishes;
- the possibility for relatives to meet the SPCT professionals.

Integration with the Haematology teams will be planned through the whole disease trajectory. Although we do not have a specific structure for liaising with haematologists, meetings and case conferences are performed periodically with specific attention to critical turning points such as periodical re-assessments, disease progression, and major modification of the therapeutic plan. Whenever possible, the two teams will have preliminary discussions to reach a shared clinical proposal for the patient to the maximum extent possible. All disagreements will be negotiated within the meetings.

### The standard PCI in the control arm

Patients randomised to the standard care arm can be seen by the SPCT at the request of their haematologist at any time. The intervention will not be standardised but planned on the patients’ needs.

The date of SPCT interventions will also be recorded for these patients.

### Statistical methods

#### Statistical analysis

The feasibility of the early integrated PC intervention will be assessed by estimating the proportion of haematologic patients who agreed to participate in the intervention and attended the first PC visit. We plan to recruit 60 study participants. Feasibility will be achieved if > 50% of patients remain in the programme 3 months after enrolment.

Secondary analyses include the quantitative evaluation of changes in the score of the four questionnaires (POS, ESAS, HADS and ECOG) within 6 months after enrolment.

Descriptive summaries of the scores will be presented by arm at each time point (baseline, 4, 8, 12, 16, 20 and 24 weeks from baseline). For each scale and for each time point, changes from baseline will also be presented with corresponding 95% confidence intervals. For each scale, differences between arms will be estimated using multi-level repeated-measures modelling adjusting for scores at baseline.

Statistical analyses will be performed by the staff of the Clinical Trials and Statistics Unit of the Azienda USL-IRCCS. To this end, the SAS System or R will be used according to availability and version in use.

### Sample size estimation

The study was designed to randomise 60 patients. Feasibility will be achieved if > 50% of patients remain in the programme 3 months after enrolment. It will be possible to assess the feasibility of the experimental intervention assuming a two-tailed type 1 error equal to 5% and a statistical power equal to 80% given the following assumptions:

1. planned statistical test: chi-squared test with 1 degree of freedom;
2. *p*-value calculation mode: exact (because of the small sample size);
3. alternative hypothesis: 75%;
4. allocation ratio: 1:1.

To reach the primary objective, the statistical test as cited in point 1 will be used according to specifications detailed in point 2; about the tested percentage, the exact two-sided 95% confidence interval will be calculated according to the Clopper-Pearson approach. Sample size was computed by using nQueryAdvisor, procedure POT0x, version 7.0.

### The qualitative analysis

The qualitative analysis will be performed as described below. Recordings of the interviews will be transcribed verbatim and then analysed using thematic analysis to explore the content and context of responses [38, 39].

Each transcript will be independently labelled by two researchers, who will reconcile differences in labelling. Throughout an iterative process, they will inductively identify a few subthemes. Finally, a third researcher will revise both the transcripts and the preliminary thematic analysis and regroup and rename some themes and subthemes with the objective of describing them by highlighting commonalities and differences between the perspectives of the three ‘actors’ involved. This revision will be discussed and emended with the other researchers involved in the qualitative analysis.

### Blinding

Allocation status cannot be blinded for the participants or trial personnel.

### Ethics

Little evidence is available in the literature concerning the beneficial effect of early palliative care in advanced haematologic cancer patients. Furthermore, patients randomised to standard treatment receive the same treatment as they would have had if they had not entered this trial, including SPCT consultation at the request of their haematologist. These palliative interventions will be reported in a case report file.

The protocol has been approved by the local ethics committee (the Ethics Committee of the Area Vasta Emilia Nord, No. 2018/0056350 of 18 May 2018) as *EPC-EMA1* and has been registered at www.clinicaltrials.gov (NCT03743480).

Any protocol amendment will be submitted in ethics committee and communicated to all the participants and to clinicaltrials.gov

## Discussion

This is the first trial assessing the early integration of palliative care in patients with advanced haematological malignancies. Other prospective studies have included patients with acute leukaemia relapse [21] or patients with high-risk acute myeloid leukaemia [40].

Palliative care is a holistic approach that aims to improve quality of life in people with life-threatening illness and in their families [41]. A growing body of literature has identified significant challenges in providing palliative care in a haematological setting [19, 42]. Barriers for palliative care integration include difficulty in prognostication by haematologists, too little research specifically on haematological cancer patients’ needs, and misperceptions about palliative care as end-of-life care [13–15].

Different haematologic diseases express different needs, but there are basic palliative care needs in all hematologic populations examined in many studies. The call for a new model of integration between palliative care and haematologic service is strong [16, 22]; some authors recommend such care just from the beginning of an advanced disease [17, 18], while other authors advocate its provision according to the patients’ needs [19, 20].

Evidence supporting an effective model of integration is missing. In clinical settings, palliative care is usually offered by haematologists in the last days/weeks of life or according to symptom burden, for example, in leukaemia patients at high risk of relapse or who have undergone transplant [10, 11, 23–26].In this trial, we will test the feasibility of an integrated palliative care approach starting when the haematologist decides to propose the last active treatment to the patient, according to his/her clinical judgement. We decided to test this criterion because it is very simple to understand, not prognosis linked and, at the same time, able to intercept a wide range of patients and needs. Moreover, the inclusion criterion (judged by the hematologists) “the last active treatment” will include incurable patients.

The hematologist specialists underestimate PC needs and are reluctant to refer patients to a PC service. These problems, in our opinion make this population more homogenous than we could think according to the haematologic diagnosis.

We therefore decide to include different onco-hematologic diseases with different trajectories and characteristics but with the same palliative care unmet need.

The feasibility of this approach requires that we enrol at least 60 patients and that more than 50% of them be followed by the palliative care team for at least 24 weeks. Failure can derive from multiple reasons: a low rate of recruitment because either the haematologists or the patients do not believe in the effectiveness of the intervention; or a low proportion of patients followed for at least 24 weeks because the patients refuse to go on or because the intervention starts too late in the course of the disease.

Conversely, if the trial shows that this approach is feasible – more than 50% of patients are followed by the palliative care team for at least 24 weeks – the impact on quality of life can be potentially relevant, considering that the seminal Temel trial [43] was designed to assess the impact of an integrated PCI at 12 weeks after randomisation.We establish 24 weeks as integrated assistance length and, on the other hand, we include as inclusion criterion “life expectancy > one month”; is an attempt to avoid enrollment of end-of-life hematologic patients considering that literature showed an difficult in prognosis by hematologists.

The trial will include integrated qualitative data analysis to deeply explore the strengths and weaknesses of the proposed approach from patients’ and professionals’ point of view; this methodology can give essential information on feasibility and acceptability, especially for vulnerable people, such as those in palliative care, data that are difficult to obtain with questionnaires or quantitative data assessment. Moreover, the qualitative approach can provide useful information for identifying the “active ingredients” of the PCI.

Regardless, this study, independently of the results, can provide useful information for modelling the most effective integrated PCI in advanced haematological cancer patients.

## Data Availability

Not Applicable.

## Abbreviations

ECOG: Eastern Cooperative Oncology Group
EPC: Early Palliative Care
ESAS: Edmonton Symptom Assessment Scale
HADS: Hospital Anxiety and Depression Scale
PC: Palliative Care
PCI: Palliative Care Intervention
POS: Palliative Care Outcome Scale
SPCT: Specialist Palliative Care Team
